# Elucidating the Pharmacological Properties of *Zingiber officinale* Roscoe (Ginger) on Muscle Ageing by Untargeted Metabolomic Profiling of Human Myoblasts

**DOI:** 10.3390/nu15214520

**Published:** 2023-10-25

**Authors:** Nur Fatin Nabilah Mohd Sahardi, Faizul Jaafar, Jen Kit Tan, Mariam Firdhaus Mad Nordin, Suzana Makpol

**Affiliations:** 1 Department of Biochemistry, Faculty of Medicine, Level 17, Preclinical Building, Universiti Kebangsaan Malaysia, Jalan Yaacob Latif, Bandar Tun Razak, Cheras, Kuala Lumpur 56000, Malaysia; 2 Jeffrey Cheah School of Medicine and Health Sciences, Monash University Malaysia, Jalan Lagoon Selatan, Bandar Sunway 47500, Selangor Darul Ehsan, Malaysia; 3 AM Zaideen Ventures Sdn. Bhd., Kuala Lumpur 54100, Malaysia

**Keywords:** ginger (*Zingiber officinale* Roscoe), myoblast, muscle ageing, metabolomic analysis

## Abstract

(1) Background: Muscle loss is associated with frailty and a reduction in physical strength and performance, which is caused by increased oxidative stress. Ginger (*Zingiber officinale* Roscoe) is a potential herb that can be used to reduce the level of oxidative stress. This study aimed to determine the effect of ginger on the expression of metabolites and their metabolic pathways in the myoblast cells to elucidate the mechanism involved and its pharmacological properties in promoting myoblast differentiation. (2) Methods: The myoblast cells were cultured into three stages (young, pre-senescent and senescent). At each stage, the myoblasts were treated with different concentrations of ginger extract. Then, metabolomic analysis was performed using liquid chromatography-tandem mass spectrometry (LCMS/MS). (3) Results: Nine metabolites were decreased in both the pre-senescent and senescent control groups as compared to the young control group. For the young ginger-treated group, 8-shogaol and valine were upregulated, whereas adipic acid and bis (4-ethyl benzylidene) sorbitol were decreased. In the pre-senescent ginger-treated group, the niacinamide was upregulated, while carnitine and creatine were downregulated. Ginger treatment in the senescent group caused a significant upregulation in 8-shogaol, octadecanamide and uracil. (4) Conclusions: Ginger extract has the potential as a pharmacological agent to reduce muscle loss in skeletal muscle by triggering changes in some metabolites and their pathways that could promote muscle regeneration in ageing.

## 1. Introduction

The world’s ageing population continues to rise at an unprecedented rate. According to Roberts et al. [1], the number of individuals aged 65 years old and above is expected to increase to 1.6 billion by the year 2050. Ageing is characterised by changes in physiological function and a decrease in the immune system, which later results in age-associated diseases such as diabetes, cancer, cardiovascular disease and sarcopenia [2]. Muscle loss, also known as sarcopenia, is one of the major diseases usually associated with ageing people aged 60 years old and above [3]. Sarcopenia is depicted by three characteristics, which are loss of muscle mass, decrease in muscle strength and decline in physical activities [3]. Deterioration in muscle later can contribute to a higher chance of frailty, limit physical activities and increase in the disability among elderly [4].

Oxidative stress is one of the major factors that contribute to muscle loss [5]. Oxidative stress occurs when there is an imbalance between reactive oxygen species (ROS) and antioxidants in the body [6]. ROS consist of superoxide radical (O_2_·^-^), hydroxyl radical (·OH), hydrogen peroxide (H_2_O_2_), peroxyl radical (ROO.), hydroperoxyl radical (HOO.) and ozone [7]. In general, muscles consume a higher level of oxygen as compared to other organs, which later leads to the existence of high oxidative stress [8]. The presence of a high level of ROS in muscle contributes to the dysfunction of mitochondria and damage in macromolecules (DNA, protein and lipid) and induces cell apoptosis [9]. It also causes an imbalance between protein synthesis and breakdown, which then leads to muscle loss and atrophy [10].

However, this problem can be prevented by practicing a healthy lifestyle. Muscle loss in the older population can be reduced through exercise and the practice of a healthy eating style [11,12]. Tohma et al. [13] have reported that antioxidant-rich food can reduce the level of oxidative stress as well as prevent diseases related to oxidative stress. Ginger (*Zingiber officinale* Roscoe) is one of the medicinal herbs that can act as an antioxidant agent [14]. The ginger plant comes from the family *Zingiberaceae* and genus *Zingiber.* This plant can be found around the subtropical area, tropical Asia, Africa, east Asia, China and India [15]. Ginger extract is not only used as a spice in cooking but also as traditional medicine for many types of diseases including cough, vomiting, nausea and digestive treatment [16]. Ginger consists of several active compounds such as 6-gingerol, 6-shogaol, 10-gingerol, 8-shogaol and paradols [15], which exhibited various types of therapeutic effects. Previous studies have reported that this ginger extract has been proven to act as an antibacterial [17], anticancer [18], anti-inflammatory [19] and antioxidant agent [14].

Metabolomics study is one branch of the omics study that has been used for elucidating the metabolome changes in a complex process. Meanwhile, the metabolite is referred to as the end product of cellular processes, which are affected by lifestyle, environment and genetic changes. Several studies have reported metabolomic changes in aged skeletal muscle. A study performed by Uchitomi et al. [20] reported that there were several metabolism changes that occurred in aged skeletal muscle, including glucose metabolism, phospholipid metabolism and polyamine metabolism. These changes have contributed to the loss of skeletal muscle mass and function in aged mice. Dabaj et al. [21] found that seven metabolites including glycerophosphocholine and adenosine triphosphate were downregulated in the Duchenne biopsies as compared to the control group. Meanwhile, 27 metabolites were upregulated in Duchenne biopsies, which included phosphatidylcholines, phosphatidic acids, sphingomyelin and phosphatidylserine. The dysregulation of these metabolites was related to energy and phospholipid metabolism. In another finding, they reported that nicotinamide riboside supplementation has enhanced the aged human skeletal muscle NAD^+^ metabolome and decreased an inflammatory signature and transcriptomic changes related to mitochondria [22]. Ohmura et al. [23] mentioned that strenuous exercise upregulated some metabolites associated with the tricarboxylic acid cycle and glycolytic pathway in skeletal muscle. However, the effect of ginger as an antioxidant agent on the metabolic pathways of aged skeletal muscle has still not been reported yet. Our previous study showed that ginger improved myoblast differentiation to myotube in culture [24]. Thus, this study aimed to determine the effect of ginger extract on the metabolic pathways in the myoblast cells for elucidating the mechanism involved and its pharmacological properties in the promotion of myoblast differentiation.

## 2. Materials and Methods

### 2.1. Cell Culture and Replicative Senescence

Primary human myoblasts (human skeletal muscle myoblast; HSMM), which were derived from a 17-year-old female, were used in this study. The human myoblast was purchased from Lonza (Walkersville, MD, USA). Myoblast cells were maintained in skeletal muscle growth media-2 (SkGM-2 medium) that consisted of skeletal muscle basal medium (SkBM), growth medium and complete culture media (CCM). SkGM-2 contained L-glutamine, fetal bovine serum, epidermal growth factor (HeGF), dexamethasone and gentamicin/amphotericin B (Lonza, Walkersville, MD, USA). Cells were trypsinised when the cell population reached 70–80% confluency. Prior to incubation in a humid atmosphere at 37 °C containing 5% carbon dioxide (CO_2_), the cells were cultivated at 5000–7500 cells/cm^2^ and the medium was warmed to 37 °C. The population doubling (PD) was calculated for each passage by using the formula:

log (N/n) log^2^ (1)

N: number of cells at the time passage; n: number of cells initially plated. The cells were divided into three groups, which were young cells with population doubling (PD) 14, pre-senescent cells with PD 18 and senescent cells with PD 21.

### 2.2. Ginger (Zingiber officinale Roscoe) Extract Preparation and Treatment Protocol

*Zingiber officinale* Roscoe (ginger) was extracted using the subcritical water extraction method to obtain the standardised ginger extract (UTM, Malaysia). The optimum condition for the extraction method was 130 °C for 30 min with a solvent-to-solid ratio of 28/2 mL/mg [25]. The stock solutions for ginger extract were freshly prepared in water at a concentration of 10 mg/mL and kept for not more than one month at −20 °C. Ginger extract was diluted with complete culture media (CCM) into a series of concentrations, which were 50, 100, 200 and 300 µg/mL. Different ages of cells were treated with various dosages of ginger extract. Young cells (PD14) were treated with 0, 50, 200 µg/mL ginger extract, pre-senescent cells (PD 18) at 0, 50, 300 µg/mL and senescent cells (PD 21) at 0, 100, 300 µg/mL These dosages were chosen based on the result of a cell viability assay from our previous study [26].

### 2.3. Sample Preparation

The myoblast cells (~6 × 10^5^) were seeded in a 100 mm plate for 24 h. After 24 h, the treated groups were treated with 6-gingerol or 6-shogaol for 24 h. Meanwhile, for the control group, the medium was replaced with the new medium. Once the treatment was completed, the media were discarded and the cells were washed with 5 mL of cold NaCl 0.9% twice and discarded. Then, 80% methanol: H_2_O solution with ratio 4:1 was added into the plate with cells. The plate was incubated at −80 °C for 15 min. The cells were then scrapped and transferred into microcentrifuge tubes. The cells were centrifuged at 20,000× *g* and 4 °C for 10 min. The supernatant was transferred into a new microcentrifuge tube and dried up using a speedvac. The dried metabolites were kept at −80 or reconstituted in 100% methanol for liquid chromatograph mass spectrometry (LCMS) analysis.

### 2.4. Metabolomic Analysis by UHPLC-MS/MS

The dried metabolite extracts were reconstituted with water and filtered with 0.22 µm regenerated cellulose membrane. Sample reconstitution was normalised to its protein concentration at 0.5 µg/µL protein for every 100 µL of reconstitution buffer. Water was used as a blank sample. A quality control (QC) sample was prepared by pooling 4 µL aliquot from each sample into 1 tube. Each group consisted of 3 biological replicates and was arranged randomly between 2 QC samples. The QC sample was injected at every 9 injections of samples.

Untargeted metabolomics analysis was performed via the Ultra High Performance Liquid Chromatography (UHPLC) system (Dionex Ultimate 3000, Thermo Scientific, Waltham, MA, USA) and Orbitrap-MS (Q-Exactive HF, Thermo Scientific) as described previously [27]. The C18 column (Synchronis, Thermo Scientific) of 100 mm × 1.7 µm was heated at 55 °C, which was run at a flow rate of 0.45 mL/min. Mobile phase A and B solvents were water and acetonitrile containing 0.1% formic acid each. The elution gradient was set at 0.5% B for 1 min, 0.5 to 99.5% B in 15 min, 99.5% B for 4 min and 99.5 to 0.5% B in 2 min. The sample was injected at 2 µL. MS1 resolution was fixed at 60,000, while MS2 was at 15,000 and stepped normalised collision energy (NCE) at 20, 40 and 60 arbitrary units. After the completion of the positive ionisation mode, data acquisition was repeated for the negative ion mode.

### 2.5. Statistical Analysis

Mass spectrometry (MS) raw data were pre-processed for peak detection, alignment, blank subtraction and annotation using Compound Discoverer 2.0 (Thermo Scientific). Metabolite identification was performed by Compound Discoverer based on the mzCloud database that was incorporated into the software. Only metabolites with accurate mass ≤ 5 ppm and MS2 spectrum match > 70% were reported as confident annotations, as described previously [28] Molecular features with signal intensity, retention time and molecular weight were exported into a CVS file for statistical analysis. MetaboAnalyst 5.0 software was used to identify the differentially expressed metabolites among groups. The univariate *t*-tests and fold change were used, and a false discovery rate (FDR) less than 0.05 was considered statistically significant [29]. Pathway analysis of the significant metabolites was also determined by using MetaboAnalyst. The pathway was considered significant and impactful when *p*-value < 0.05 with impact value > 0.1.

## 3. Results

### 3.1. Differential Metabolomic Analysis between Control and Treated Myoblast Group

Principle component analysis (PCA) of young control myoblasts (YC) against pre-senescent control myoblast (PSC) generated a 56.3% variation in the negative mode, whereby the PC1 and PC2 scores were 40.9% and 15.4% (Supplementary Materials, Figure S1(1a)). Meanwhile, the PCA score plot for positive mode for this group comparison displayed a variation of 63.0%, with 45.3% of PC1 and 17.7% of PC2 (Supplementary Materials, Figure S1(1b)). The PCA score plots showed good separation between groups.

The PCA score plot between the YC and senescent control myoblasts (SC) showed a 54.1% variation for negative mode, with 40.8% of PC1 and 13.3% of PC2 (Supplementary Materials, Figure S1(2a)). For positive mode, the PCA score plot between these two groups of myoblasts showed a 59.2% variation, which comprises 46.2% for PC1 and 13.1% for PC2 (Supplementary Materials, Figure S1(2b)). Both PCA score plots for the negative and positive modes displayed an overlapping between SC and YC groups.

The PCA score for YC against young myoblast treated with 50 µg/mL ginger extract (YT50) generated a 51.7% variation for negative mode, whereby the PC1 score was 39.9% and the PC2 score was 11.8% (Supplementary Materials, Figure S1(3a)). The PCA score for positive mode showed a 63.2% variation with, PC1 47.6% and PC2 15.6% (Supplementary Materials, Figure S1(3b)). Both PCA displayed an overlapping in negative and positive modes. The PCA score between YC and young myoblast treated with 200 µg/mL ginger extract (YT200) showed a 53.1% variation for the negative mode (Supplementary Materials, Figure S1(4a)), with 41.3% for PC1 and 11.8% for PC2. The positive mode for these two groups exhibited 63.8% variation, with 46.0% for PC1 and 17.8% for PC2 (Supplementary Materials, Figure S1(4b)). There was overlapping for the PCA score plot in both positive and negative modes.

The PCA for PSC against pre-senescent myoblast treated with 50 µg/mL ginger extract (PST50) displayed a 54.4% variation for negative mode, with 42.7% of PC1 and 11.8% of PC2 (Supplementary Materials, Figure S1(5a)). For positive mode, this group comparison generated a 62.8% variation, with 52.4% of PC1 and 10.4% of PC2 (Supplementary Materials, Figure S1(5b)). The PCA score showed an overlapping between PSC and PST50. The PCA score of negative mode for group PSC against pre-senescent myoblast treated with 300 µg/mL ginger extract (PST300) presented a 57.8% variation, which consisted of 45.3% of PC1 and 12.5% of PC2 (Supplementary Materials, Figure S1(6a)). The PCA score for positive mode for PSC against PST300 exhibited 63.2% variation, whereby 53.9% was for PC1 and 9.3% was for PC2 (Supplementary Materials, Figure S1(6b)). The PCA score for negative mode showed an overlapping, while positive mode only displayed a slight overlapping between the PSC and PST300 groups.

The PCA score plot for SC and senescent myoblast treated with 100 µg/mL ginger extract (ST100) generated a 46.2% variation for negative mode (32.7% for PC1 and 13.5% for PC2) (Supplementary Materials, Figure S1(7a)). For positive mode, the comparison between these groups showed a 63.3% variation, with 49.7% of PC1 and 13.65% of PC2 (Supplementary Materials, Figure S1(7b)). There was overlapping in both modes. Meanwhile, the PCA score for negative mode between group SC against senescent myoblast treated with 300 µg/mL ginger extract (ST300) displayed a 51.3% variation, which comprises 36.0% of PC1 and 15.3% of PC2 (Supplementary Materials, Figure S1(8a)). The comparison between these groups generated a 62.6% variation in PCA score for positive mode, whereby 46.4% was for PC1 and 16.2% was for PC2 (Supplementary Materials, Figure S1(8b)). Both modes showed an overlapping between groups.

### 3.2. Comparison of Metabolites Profile in Control and Ginger-Treated Myoblast Cell

The effect of ageing on metabolites profile was determined based on the comparison between YC, PSC and SC groups. A comparison between PSC and YC showed 36 significant metabolites (Table 1), which were differentially expressed. Meanwhile, a comparison between SC and YC groups generated 18 significant metabolites. Only changes of 10 metabolites were similar in these 2 comparisons (PSC vs. YC and SC vs. YC). Out of the 10 metabolites, only choline with an FDR value less than 0.05 showed a different pattern in these comparisons. Choline was downregulated by 1.25-fold in PSC against YC, while for comparison between SC and YC, this metabolite was upregulated by 1.21-fold in SC. The most upregulated metabolite in the PSC group vs. YC group was adenosine (32.748-fold) followed by adenine (22.804-fold). In the SC group vs. YC group, the most upregulated metabolite was NADH with 2.58-fold. However, the most downregulated metabolite in both comparisons was adipic acid, whereby the fold change was 21.80 and 17.95 for PSC vs. YC and SC vs. YC, respectively.

For the effect of ginger on myoblast cells, our study found that comparison between YC and YT50 showed nine significant metabolites, whereby five metabolites were upregulated and another four metabolites were downregulated (Table 2). For the comparison between YT200 and YC, 14 metabolites were significantly different, consisting of 10 upregulated metabolites and 4 downregulated metabolites. Changes of five metabolites were similar in both comparisons (YT50 vs. YC and YT200 vs. YC), which were 2,2′-methylenebis (4-methyl-6-tert-butylphenol), adipic acid, (8)-shogaol, bis(4-ethylbenzylidene) sorbitol and valine. The most upregulated metabolite in YT50 vs. YC and YT200 vs. YC was (8)-shogaol with fold values 8.21 and 36.1, respectively, followed by valine with fold values 1.37 and 1.47, respectively. Both comparisons also showed a similar change for some of the downregulated metabolites such as adipic acid, bis(4-ethylbenzylidene) sorbitol and 2,2′-methylenebis(4-methyl-6-tert-butylphenol). In YT50 vs. YC, adipic acid, bis(4-ethylbenzylidene) sorbitol and 2,2′-methylenebis(4-methyl-6-tert-butylphenol) were downregulated by 9.07-fold, 2.93-fold and 2.09-fold, respectively. However, adipic acid was more downregulated in YT200 vs. YC by 15.15-fold.

For the pre-senescent myoblast groups treated with ginger extract, 11 metabolites were significant in PST50 vs. PSC groups, while 12 significant metabolites were identified in PST300 vs. PSC groups (Table 3). In the PST50 vs. PSC comparison, seven metabolites were upregulated and four metabolites were downregulated. Meanwhile, in the PST300 vs. PSC comparison, eight metabolites were upregulated and four metabolites were downregulated. However, for both comparisons (PST50 vs. PSC and PST300 vs. PSC), only three metabolites were similarly changed, whereby two metabolites were downregulated and another one was upregulated. For PST50 vs. PSC, pyridoxine, phenylalanine and bis(4-ethylbenzylidene) sorbitol were found to be the most upregulated metabolites, with an increase of 3.05-fold, 1.49-fold and 1.482-fold correspondingly. Glutathione (3.05-fold) and citric acid (1.18-fold) were the most downregulated metabolites in PST50 vs. PSC. In PST300 vs. PSC, the most upregulated metabolite was (8)-shogaol, with an increase of 34.32-fold, followed by xanthine (1.78-fold) and uric acid (1.70-fold).

For senescent cells comparison, there were eight significant metabolites displayed in ST100 against SC groups, consisting of four upregulated metabolites and four downregulated metabolites (Table 4). (8)-Shogaol and octadecanamide were the most upregulated in ST100 vs. SC. In contrast, glutathione was the most downregulated metabolite in ST100 vs. SC, followed by uric acid. In ST300 against SC groups, 20 significant metabolites were recognised. In total, 11 of them were upregulated and 9 were downregulated. (8)-Shogaol, adenosine and adenine were displayed as the most upregulated metabolites in this group. Meanwhile, uric acid, 4-oxoproline and pyroglutamic acid were found to be the most downregulated metabolites. Between the ST100 vs. SC and ST300 vs. SC comparisons, there were five similar metabolites, which were uric acid, (8)-shogaol, carnitine, octadecanamide and uracil. Uric acid was downregulated by 2.51-fold in ST100 against SC and 2.58-fold in ST300 against SC. Meanwhile, (8)-shogaol, octadecanamide and uracil were upregulated in both ST100 vs. SC and ST300 vs. SC comparisons.

### 3.3. Biochemical Pathways Analysis of Muscle Metabolomes in Control and Myoblast-Treated Groups

**Figure 1:**
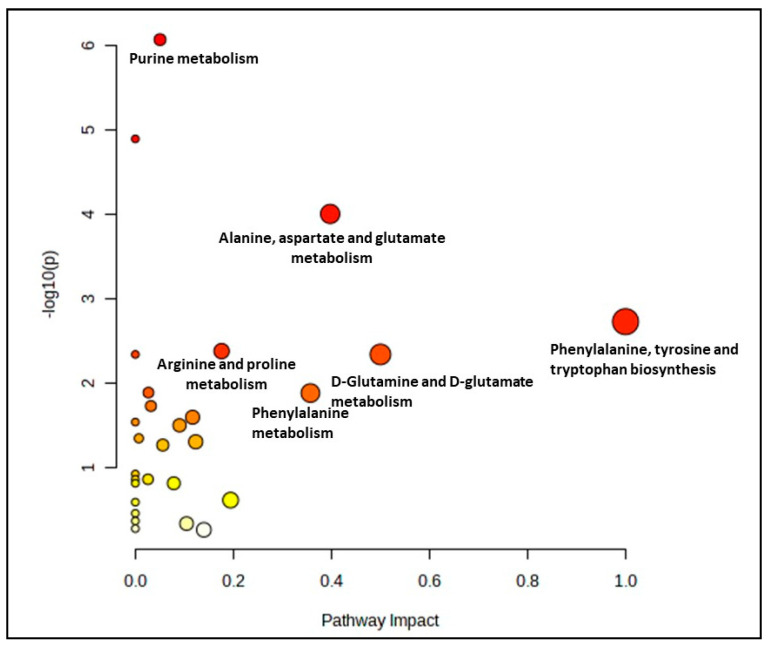
Biochemical pathway analysis of significant metabolites profile for myoblast control groups (young, pre-senescent and senescent cells).

A comparison between the control groups (young, pre-senescent and senescent) generated 28 biochemical pathways, as shown in Figure 1. However, only seven pathways were significant and impactful for this group comparison, which were the alanine, aspartate and glutamate metabolism, phenylalanine, tyrosine and tryptophan biosynthesis, arginine and proline metabolism, D-glutamine and D-glutamate metabolism, phenylalanine metabolism, arginine biosynthesis and citrate cycle (TCA cycle) pathway. (Supplementary Materials, Table S1).

**Figure 2:**
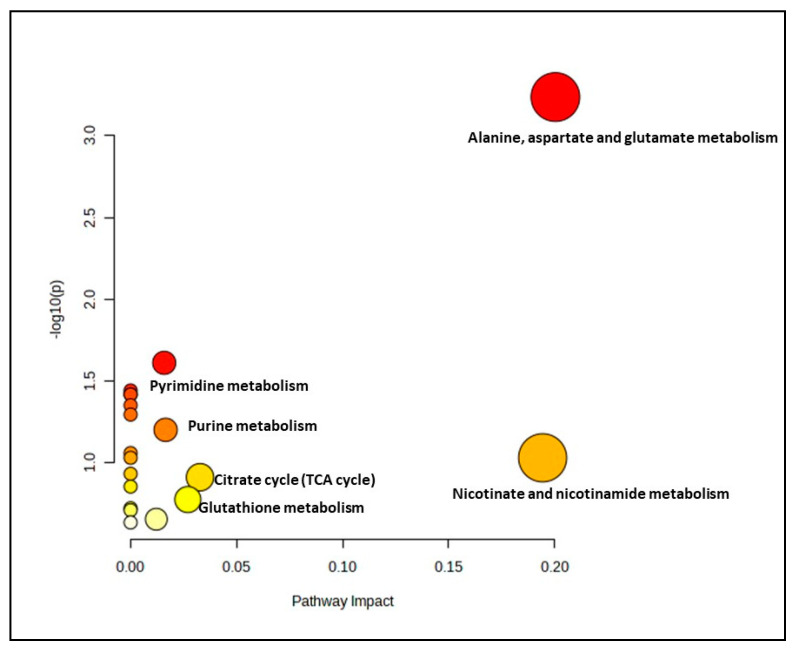
Biochemical pathway analysis of significant metabolites profile for the young myoblast treated with or without ginger extract.

The effect of ginger extract on the metabolic pathway was evaluated by comparing the metabolome expression in myoblasts treated with and without ginger extract in young, pre-senescent and senescent groups. A significant metabolite profile in the young treatment groups (YT50 or YT200 vs. YC) determined 19 biochemical pathways (Figure 2 and Supplementary Materials, Table S2). However only one pathway identified for this group as a significant and impactful pathway which was alanine, aspartate and glutamate metabolism pathway.

**Figure 3:**
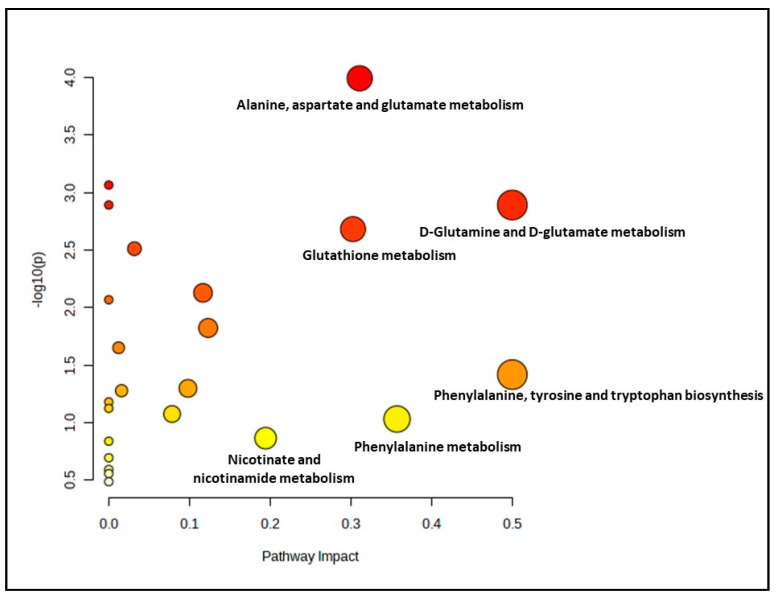
Biochemical pathway analysis of significant metabolites profile for the pre-senescent myoblast treated with or without ginger extract.

Meanwhile, for the pre-senescent myoblast groups treated with ginger extract, 23 biochemical pathways were identified. 6 out of 23 pathways were evaluated as significant and impactful pathways in these groups, as shown in Figure 3 and Supplementary Materials, Table S3. The significant and impactful pathways were alanine, aspartate and glutamate metabolism, D-glutamine and D-glutamate metabolism, arginine biosynthesis, citrate cycle (TCA cycle), phenylalanine, tyrosine and tryptophan biosynthesis as well as glutathione metabolism pathways.

**Figure 4:**
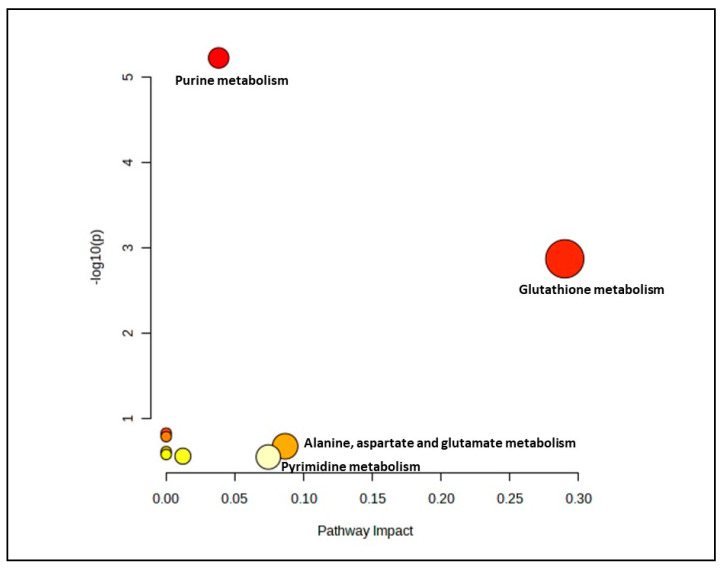
Biochemical pathway analysis of significant metabolites profile for the senescent myoblast groups treated with or without ginger extract.

In the senescent myoblast groups treated with gingered extract, only 14 biochemical pathways were generated (Figure 4). However, only two pathways which were glutathione metabolism and alanine, aspartate and glutamate metabolism pathways were identified as significant and impactful pathway (Supplementary Materials, Table S4).

## 4. Discussion

Muscle loss is a common problem that occurs among the older population, and this can be reflected by the changes in various types of metabolites. Therefore, the determination of metabolites in aged (senescent) skeletal muscle cells could be useful for understanding the mechanism of muscle loss among the older population. In this study, the comparison between the control groups at a different PD of myoblast cells has been performed to determine the effect of ageing on the metabolite changes of skeletal muscle. The current study showed that some metabolites including adipic acid, succinic acid, leucine and methionine were downregulated in both the pre-senescent and senescent control groups as compared to the young control group. After treatment with ginger extract, metabolites such as succinic acid, leucine, valine, carnitine and glutamine were changed, which may indicate the positive effect of ginger treatment in aged myoblasts. Our previous study showed that ginger treatment contributes to the prevention of cellular senescence and the promotion of myoblast regenerative capacity [24].

Adipic acid is one of the products of lysine metabolism, which come from the catabolism of lysine, which involves fatty acid oxidation [30]. Lysine could be catabolised into saccharopine and then converted into aminoadipic acid by enzymes L-lysine ketoglutarate reductase and saccharopine dehydrogenase [31]. Our current finding showed that adipic acid was downregulated in pre-senescent and senescent control groups, which was similar to the previous study. Munasinghe et al. [32] reported that ageing resulted in a significant reduction in adipic acid in *C. elegans*. This is because during ageing, there was impaired fatty acid beta oxidation, which may lead to decreased adipic acid production. Wanders et al. [33] showed that the defect in the oxidation of long-chain fatty acid has a risk of developing cardiac and skeletal muscle abnormalities such as cardiomyopathy and arrhythmias. The level of adipic acid was also decreased in the young treatment group. This may also indicate the impairment in fatty acid oxidation. However, the downregulation of adipic acid in the treatment group was lower than in the control group, which may indicate the positive effect of ginger on fatty acid oxidation impairment.

Succinic acid or succinate is an intermediate in a tricarboxylic acid (TCA) cycle, which generates energy in the form of ATP [34]. A previous study reported that this metabolite was essential in regulating mitochondrial function as well as reducing the level of oxidative stress and inflammation [35]. Succinic acid has potential in skeletal muscle fiber-type transition by inducing a conversion from fast-twitch to slow-twitch fibers [36]. This transition could be seen through the enhancement of calcium and some vital transcriptional factors including MEF2, calcineurin and NFATc1, which are essential in skeletal muscle fiber switching. Succinic acid also increases the expression of PGC-1α and mitochondrial content, thereby improving oxygen uptake in skeletal muscle cells [36]. In another study, it was found that this metabolite could activate the G-protein-coupled receptor SUNCR1 [36]. The activation of SUNCR1 by succinic acid leads to the elevation of platelet, hemoglobin and neutrophils as well as increased immunity. The results of our current study showed that this metabolite was downregulated in pre-senescent and senescent control groups as compared to the young control group. This may occur due to ageing effects, whereby the ageing process could lead to mitochondrial dysfunction and higher levels of oxidative stress and inflammation [6,37]. The ability of ginger to improve mitochondrial function, improve of platelet, hemoglobin, and neutrophils as well as increase immunity could be seen via upregulation of succinic acid in the young and pre-senescent myoblast cells after treatment with ginger extract. This was in line with previous studies that found ginger could promote mitochondrial biogenesis [38] and the immune system [39].

Another important metabolite found to be significant in this study was uric acid. Uric acid is the final product of purine metabolism and could exhibit antioxidant properties [40]. Purine metabolism is essential for the synthesis of building blocks of DNA and RNA, providing energy as well as promoting cell survival and proliferation [40]. A previous study has reported a positive association between uric acid and muscle mass in the older population [41]. They suggested that uric acid could be a protective factor for muscle strength in the elderly. However, a study by Nahas et al. [42] found that uric acid was not connected with the appendicular muscle mass of young and middle-aged adults. The different results found between young and old adults may be due to the effect of uric acid on the muscle, which varies according to its composition and is known to be altered during ageing. As individuals age, the decline in fast-twitch muscle fibers exceeds the reduction observed in slow-twitch muscle fibers, which exhibit relatively low sensitivity to acute oxidative stress [43].

Furthermore, the positive connection between uric acid and muscle function identified in elderly individuals could be influenced by uric acid’s ability to act as an antioxidant within the central nervous system, particularly against motor neurons’ death during ageing [44]. In another study, a positive correlation was observed between uric acid level and handgrip strength in the aged population [45]. The low uric acid level contributed to the low handgrip strength, which represents the overall muscle strength in the elderly population. This was in line with this current study, which found that uric acid was downregulated in the pre-senescent control group. Meanwhile, the senescent control group did not show any changes. A previous study found that uric acid level in skeletal muscle of aged mice remained constant from 12–30 months [46]. This may be due to the decline of uricase activity during ageing. The ginger treatment in myoblasts caused an upregulation of uric acid in pre-senescent myoblasts treated with ginger at a high concentration. The possible mechanism that explained the beneficial impact of uric acid on muscle and bone might be due to the antioxidant capacity of uric acid itself [47]. However, uric acid was downregulated in senescent myoblasts treated with ginger at both concentrations. This may be due to the properties of ginger itself as a xanthine oxidase inhibitor [48]. Xanthine oxidase is involved in the production of uric acid, whereby uric acid levels decrease together with the reduction of xanthine oxidase [49].

Guanidinosuccinic acid (GSA) is a derivative of L-arginine and a precursor of creatine [50]. The findings of this current study showed that GSA was downregulated in the control myoblast groups with increasing PD. This is because the GSA did not link directly to skeletal muscle regeneration, but it could contribute to the many types of metabolic disorders. A previous study has reported that this metabolite accumulates in patients with metabolic disorder [51]. Arnold et al. [52] found that GSA was elevated in patients with chronic kidney function, and this metabolite is also considered a uremic toxin [52]. GSA would target specific receptors in brains and could function as neurotoxins or excitotoxins. Their binding on the N-methyl-D-aspartate (NMDA) receptor leads to the overstimulation of certain neurons and enhanced intracellular level of ionised calcium before increasing the level of ROS, which could contribute to apoptosis and cell death [53].

Leucine and valine are essential amino acids for muscle protein synthesis and maintenance. A previous study has suggested that leucine was altered in the aged muscle, which was similar to the findings of the present study [54]. In this study, leucine was downregulated in the control groups with increasing PD, maybe due to the ageing effect. Opazo et al. [55] found that the level of leucine was reduced in sarcopenic individuals as compared to the control group. This was supported by another study, which found that the older adults who were supplemented with leucine improved their muscle strength and function [56]. Chae et al. [57] reported that there was a positive association between supplementation of branched-chain amino acids (BCAA), which include leucine and valine, and skeletal muscle index among middle-aged and pre-elderly adults. This may be due to the ability of leucine and valine themselves to stimulate muscle synthesis. Muscle protein synthesis by leucine and valine could be seen through the activation of the mechanistic target of mTORC1 and the activated translational process from mRNA to protein [58,59]. Meanwhile, Petrocelli et al. [60] reported that the administration of leucine to aged mice suppressed muscle atrophy by increasing the satellite cells and collagen remodeling. These changes occur due to the increment pathway that is related to oxidative phosphorylation as well as the decreased inflammatory pathway. The positive effect of ginger treatment in inducing skeletal muscle synthesis could be observed through the upregulation of valine in the young ginger-treated group as well as leucine in the pre-senescent myoblast ginger-treated group.

The findings of the current study also showed a downregulation of methionine in the control groups with increasing PD. Methionine is also considered a vital amino acid in protein synthesis. It carries cellular functions such as the regulation of gene expression and antioxidant defense [61]. This metabolite enhanced protein that is related to the mitochondrial function such as the electron transport chain, TCA cycle and respiration [62]. The expression of genes associated with this metabolite was altered in the muscle of aged rats [63]. Methionine restriction could increase lifespan by postponing cellular senescence [64]. One of the possible mechanisms of methionine restriction is by influencing insulin/IGF-1 and mTORC1 signaling [65]. By restricting methionine, the mTORC1 did not activate and then inhibited the suppression of autophagy [66]. However, all the ginger-treated groups did not show any significant effect on this metabolite.

Furthermore, the downregulation of octadecanamide in the control groups with increasing PD also reflected the ageing effect. In contrast, ginger supplementation on myoblast cells has upregulated this metabolite in the senescent group. Octadecanamide is a fatty acid amide also known as oleamide. This metabolite has been proven to increase anti-inflammatory activity and microglial phagocytosis [67]. A study performed by Zhang et al. [68] found that exercise leads to the increment of octadecanamide in skeletal muscle, which suggested that this metabolite may act as a signaling molecule in skeletal muscle. Supplementation of octadecanamide has been proven to restore tibialis anterior muscle atrophy in mice by activating Akt/mTOR signaling [69].

Pyridoxine, also known as vitamin B6, is an essential co-factor for various biochemical reactions in cellular metabolism involving synthesis and catabolism of amino acids, neurotransmitters and fatty acids as well as reducing the level of reactive oxygen species [70]. Due to the ageing effect, this current study showed that pyridoxine was downregulated in the control groups with increasing PD. Surprisingly, after 50 µg/mL ginger extract treatment, pyridoxine was upregulated in the pre-senescent group. A study performed by Kumar et al. [71] reported that pyridoxal, one of the metabolites of vitamin B6, was upregulated during myoblast differentiation. This upregulation contributes to the modulation of myokines, Nrf2-related factors, myogenin and HSP60 [72]. Thus, the upregulation of pyridoxine found in this study may also show a similar effect on myoblast differentiation, which indicated the potential of ginger in promoting skeletal muscle differentiation. In addition, this metabolite could regulate the function of muscle satellite cells together with the improvement of muscle regeneration by inducing proliferation and preventing apoptosis of satellite cells [73]. Deficiency of this metabolite will lead to the impairment of transcellular signaling between neurons and causes muscular convulsions, peripheral neuropathy and hyperirritability [74]. Zhou and Effiong [75] reported that a deficiency of pyridoxine leads to muscle spasms in patients with type 2 diabetes.

Niacinamide, also known as nicotinamide, was upregulated in the young and pre-senescent myoblasts treated with ginger extract. However, niacinamide was also increased in the senescent control group as compared to young control group, which contradicts the ginger treatment effect. This was in contrast with a previous study, which showed that nicotinamide adenine dinucleotide (NAD) decreased progressively during ageing in humans and rodents. [76]. The increase of niacinamide in senescent myoblasts found in this study may reflect the attempt to respond to the oxidative stress and reduce the production of senescence-associated secretory phenotype (SASP) in aged myoblasts. This is because niacinamide is one of the amides of vitamin B_3_ (niacin), which consists of antioxidant properties and can reduce oxidative stress as well as the SASP factor [77,78,79]. This metabolite could regulate the NF-Kβ-mediated transcription of signaling molecules via inhibition of nuclear poly (ADP-ribose) polymerase-1 (PARP-1). In previous research, it was found that nicotinamide riboside (NR) supplementation decreased the circulating inflammatory cytokines in older individuals [22]. The level of interleukin-6, interleukin-5, interleukin-2 and tumor necrosis factor-alpha (TNF-α) decreased after supplementation with NR, which may reflect the same effect of ginger treatment. Niacinamide also has been proven for reducing muscle dysfunction-related diseases. A study done by Sahin et al. [80] reported that niacinamide supplementation reduced inflammation response in rats with osteoarthritis.

(8)-Shogaol is one of the bioactive compounds in ginger, which exhibited many therapeutic effects [81]. A study done by Shieh et al. [82] found that (8)-shogaol induced apoptosis and inhibited growth in human leukemia cells by several mechanisms. Firstly, (8)-shogaol triggers the excessive production of ROS and interferes with mitochondrial functions during the initial phases of apoptosis; subsequently, it stimulates caspase-9 activation. The elevated ROS production leads to a depletion of glutathione levels, ultimately playing a role in triggering apoptosis. (8)-Shogaol could inhibit rheumatoid arthritis by reversing pathologies of rheumatoid arthritis [83]. (8)-Shogaol has been proven to inhibit IL-1β, TNF-α and IL-17 by targeting transforming growth factor β-activated kinase 1 (TAK1). In addition, (8)-shogaol exhibited an anti-inflammatory effect on DSS-induced colitis in mice by regulating NF-Kβ signaling [84]. From this study, we can observe that (8)-shogaol was upregulated in all groups of myoblast cells treated with ginger extract, indicating its positive effect particularly on improving muscle regeneration in aged skeletal muscle, as this metabolite was more upregulated in senescent myoblasts than the young myoblasts.

Glutamine is a major energy source for proliferating cells such as cancer cells and hematopoietic stem cells [85]. Glutamine is converted to glutamate in the mitochondria by glutaminase [86]. Then, glutamate is converted into α-ketoglutarate, which is the TCA cycle intermediate by glutamate dehydrogenase or by alanine or aspartate transaminase. A previous study reported that glutamine reduced muscle tissue degradation by activating satellite cells, especially after exercise [87]. In another study, it was found that glutamine improved skeletal muscle cell differentiation as well as inhibited myotube atrophy by regulating p38 MAPK [88]. Our current study showed the downregulation of glutamine in the pre-senescent control group. In contrast, the young and pre-senescent ginger-treated groups displayed an upregulation, which indicated the potential of ginger extract in promoting muscle regeneration.

**Figure 5:**
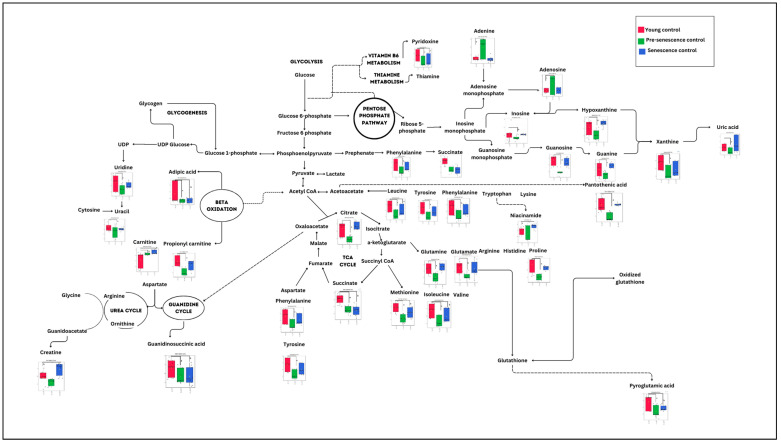
Summary of significant metabolic changes during ageing in myoblast cells. ^a^ *p* < 0.05: significantly different for pre-senescent control group vs. young control group. ^b^ *p* < 0.05: significantly different for senescent control group vs. young control group.

**Figure 6:**
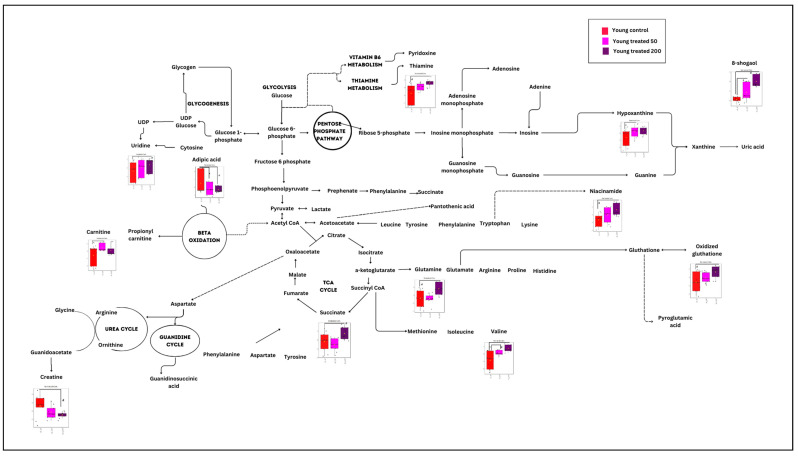
Summary of significant metabolic changes after ginger treatment in the young myoblast cells. ^c^ *p* < 0.05: significantly different for young treatment group at concentration of 50 µg/mL vs. young control group. ^d^ *p* < 0.05: significantly different for young treatment group at concentration of 200 µg/mL vs. young control group.

**Figure 7:**
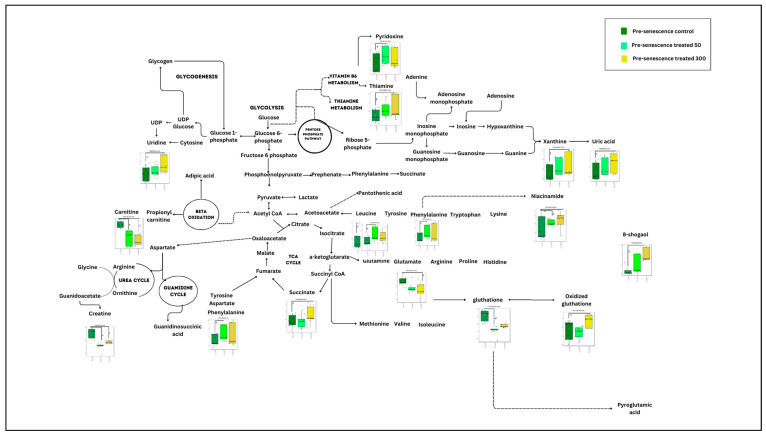
Summary of significant metabolic changes after ginger treatment in the pre-senescent myoblast cells. ^e^ *p* < 0.05: significantly different for pre-senescent treatment group at concentration of 50 µg/mL vs. pre-senescent control group. ^f^ *p* < 0.05: significantly different for pre-senescent treatment group at concentration of 300 µg/mL vs. pre-senescent control group.

**Figure 8:**
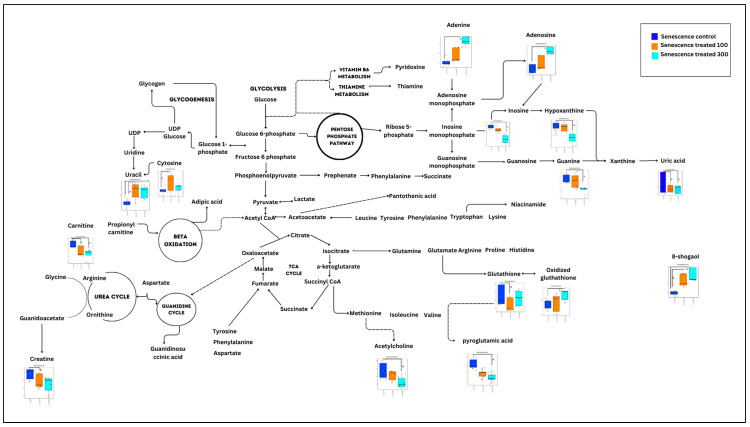
Summary of significant metabolic changes after ginger treatment in the senescent myoblast cells. ^g^ *p* < 0.05: significantly different for senescent treatment group at concentration of 100 µg/mL vs. senescent control group. ^h^ *p* < 0.05: significantly different for senescent treatment group at concentration of 300 µg/mL vs. senescent control group.

Carnitine is an amino acid derivative, which is essential in fatty acid β-oxidation. In fatty acid β-oxidation, this metabolite would transport long-chain fatty acids from the cytosol to the mitochondrial matrix [89]. The products of β-oxidation, which are a two-carbon molecule, will be used by the Krebs cycle to produce energy in the form of adenosine triphosphate (ATP) [90]. In our study, this metabolite was increased in the pre-senescent ginger-treated group at a concentration 50 µg/mL, which demonstrated the positive effect of ginger treatment. A previous study reported that carnitine suppressed the loss of skeletal muscle mass in patients with liver cirrhosis [91]. In another study, combined supplementation of L-carnitine, creatine and leucine had significantly increased muscle mass and strength through increment of mTOR protein level [92]. In our study, the senescent ginger-treated group displayed a downregulation of carnitine, which was similar to the pre-senescent and senescent control groups as compared to the young control group. One possible factor that contributed to this increment was a compensatory response to aged-related changes in energy metabolism and production. As ageing occurred, there was a decline in the mitochondrial function, which may contribute to the decrease in energy availability in the form of ATP [93]. This later leads to an increase in the demand for fatty acid oxidation to produce energy. Treatment of ginger extract in the senescent group probably did not improve the mitochondrial function during ageing. The significant metabolic changes in the control group are summarised in Figure 5. Meanwhile, the summaries of significant metabolic changes for the young, pre-senescent and senescent ginger-treated groups are shown in Figure 6, Figure 7 and Figure 8, respectively.

This is the first study highlighting the metabolic alterations in the skeletal muscle cells following ginger extract treatment. The findings of this study could provide valuable insights into the precise mechanisms through which ginger impacts muscle cells and enhancing our comprehension of its potential benefits. Nonetheless, the outcomes of this research may differ across various models, including both animal and human models, since each model displays varying metabolic responses to ginger extract. Hence, conducting additional research on metabolic changes induced by ginger extract in different models may be necessary to gain a better understanding of how ginger influences the loss of skeletal muscle in elderly individuals.

## 5. Conclusions

In conclusion, ginger treatment has contributed to the changes of some metabolites such as leucine, valine, carnitine, pyridoxine, niacinamide and glutamine, whereby most of these metabolites were downregulated due to the ageing effect. These changes may indicate the potential pharmacological properties of ginger in improving muscle regeneration and energy production of aged skeletal muscle. In future research, it may be necessary to integrate metabolomics data with genomics, transcriptomics and proteomics data to attain a more comprehensive understanding of how ginger extract affects skeletal muscle systems.

## Figures and Tables

**Table 1:** List of significant metabolites in myoblast control groups.

Mode	Metabolite	ID	MW	RT	FC	
					PSC vs. YC	SC vs. YC
neg	4-Oxoproline	METPA0228	129.04169	0.849	−2.39	−
neg	2,2′-Methylenebis (4-methyl-6-tert-butylphenol	mzc1138	340.24016	12.551	−	−3.40
neg	Adipic acid	HMDB0000448	146.05714	2.275	−21.80	−17.95
neg	Citric acid	HMDB0000094	192.0265	0.788	−1.41	−
neg	Glutamic acid	HMDB0000148/HMDB0003339	147.05221	0.603	−1.34	−
neg	Guanosine	HMDB0000133	283.09157	1.319	−1.38	−
neg	N-Acetyl-L-aspartic acid	HMDB0000812	175.04736	0.755	+1.46	−
neg	Pantothenic acid	HMDB0000210	219.11032	2.158	−2.01	−
neg	Pyridoxine	HMDB0000239	169.07315	0.826	−1.42	−
neg	Succinic acid	HMDB0000254	118.02569	0.842	−1.55	−1.58
neg	Uric acid	HMDB0000289	168.0276	0.837	−1.67	−
neg	Uridine	HMDB0000296	244.06954	0.927	−1.22	−
neg	Xanthine	HMDB0000292	152.03263	0.986	−2.05	−
pos	2-Hydroxycinnamic acid/4-Hydroxycinnamic acid	HMDB0002641/HMDB0002035	164.04743	0.934	−1.78	−
pos	Acetylcholine	HMDB0000895	145.1103	0.891	−1.18	−
pos	Adenine	HMDB0000034	135.05467	1.185	+22.80	−
pos	Adenosine	HMDB0000050	267.09665	1.242	+32.75	−
pos	Bis(4-ethylbenzylidene) sorbitol	mzc7437	414.20382	9.219	−3.93	−3.52
pos	Carnitine	HMDB0000062	161.10519	0.729	+1.27	+1.40
pos	Choline	HMDB0000097	103.10003	0.811	−1.25	+1.22
pos	Creatinine	HMDB0000562	113.05909	0.645	−1.39	−
pos	Glutamine	HMDB0003423/HMDB0000641	146.06919	0.605	−1.40	−
pos	Guanidinosuccinic acid	HMDB0003157	175.05853	0.977	−1.48	−1.45
pos	Guanine	HMDB0000132	151.04942	1.274	−1.25	−
pos	Hypoxanthine	HMDB0000157	136.03853	1.254	−1.32	−
pos	Indoleacrylic acid	HMDB0000734	187.06341	2.855	−1.84	−
pos	Inosine	HMDB0000195	268.08055	1.249	−1.54	−
pos	Leucine/Isoleucine/Norleucine	HMDB0000687/HMDB0000172	131.09468	0.98	−1.69	−1.31
pos	Methionine	HMDB0000696	149.05104	0.79	−2.12	−1.40
pos	Octadecanamide	HMDB0034146	283.28734	13.922	−1.83	−2.29
pos	Phenylalanine	HMDB0000159/METPA0264	165.07898	1.481	−1.86	−
pos	Proline	HMDB0000162/HMDB0003411	115.06359	0.701	−1.49	−
pos	Propionyl carnitine	HMDB0000824	217.13122	1.488	−1.89	−
pos	Pyridoxine	HMDB0000239	169.074	0.853	−1.59	−1.43
pos	Pyroglutamic acid	HMDB0000267	129.04268	0.852	−2.24	−1.85
pos	Tyrosine	HMDB0000158/HMDB0006050	181.07399	0.934	−1.80	−
pos	Uracil	HMDB0000300	112.02756	0.932	−1.16	−
pos	Creatine	HMDB0000064	131.06955	0.655	−	+1.26
pos	Docosanamide	HMDB0000583	339.34976	16.095	−	−2.34
pos	Erucamide	mzc282	337.33412	15.515	−	−1.96
pos	NADH	HMDB0001487	663.10909	0.943	−	+2.58
pos	Niacinamide	HMDB0001406	122.0482	0.67	−	+1.74
pos	PEG n5	mzc1819	238.14147	3.537	−	−2.58

Abbreviations: MW: molecular weight; RT: retention time; FC: fold change; YC: young cell control group; PSC: pre-senescent cell control group; SC: senescent cell control group. Symbols: +: increase; −: decrease; neg: negative; pos: positive.

**Table 2:** List of significant metabolites in young myoblast groups treated with ginger extract.

Mode	Metabolite	ID	MW	RT	FC	
					YT50 vs. YC	YT200 vs. YC
neg	2,2′-Methylenebis(4-methyl-6-tert-butylphenol)	mzc1138	340.24016	12.551	−2.09	−2.31
neg	4-Oxoproline	METPA0228	129.04169	0.849	−2.02	−
neg	Adipic acid	HMDB0000448	146.05714	2.275	−9.07	−15.15
neg	N-Acetyl-L-aspartic acid	HMDB0000812	175.04736	0.755	+1.55	−
neg	Oxidised glutathione	HMDB0003337	612.1527	0.892	−	+1.50
neg	Succinic acid	HMDB0000254	118.02569	0.842	−	+1.16
neg	Uridine	HMDB0000296	244.06954	0.927	−	+1.14
pos	(8)-Shogaol	HMDB0031463	276.1723	8.295	+8.21	+36.1
pos	Bis(4-ethylbenzylidene) sorbitol	mzc7437	414.20382	9.219	−2.93	−2.73
pos	Carnitine	HMDB0000062	161.10519	0.729	+1.30	−
pos	Hypoxanthine	HMDB0000157	136.03853	1.254	+1.20	−
pos	Valine	HMDB0000883	117.07922	0.728	+1.37	+1.47
pos	Creatine	HMDB0000064	131.06955	0.655	−	−1.18
pos	Cytosine	HMDB0000630	111.04353	0.667	−	+1.39
pos	Glutamine	HMDB0003423/HMDB0000641	146.06919	0.605	−	+1.24
pos	Niacinamide	HMDB0001406	122.0482	0.67	−	+2.13
pos	PEG n7	mzc1821	326.19378	4.316	−	+2.04
pos	Thiamine	HMDB0000235	264.10438	0.77	−	+1.47

Abbreviations: MW: molecular weight; RT: retention time; FC: fold change; YC: young cell control group; YT50: young cell treatment group at concentration 50 µg/mL; YT200: young cell treatment group at concentration 200 µg/mL. Symbols: +: increase; −: decrease; neg: negative; pos: positive.

**Table 3:** List of significant metabolites in pre-senescent myoblast groups treated with ginger extract.

Mode	Metabolite	ID	MW	RT	FC	
					PST50 vs. PSC	PST300 vs. PSC
neg	Citric acid	HMDB0000094	192.0265	0.788	−1.18	−
neg	Pyridoxine	HMDB0000239	169.07315	0.826	+1.36	−
neg	2,2′-Methylenebis(4-methyl-6-tert-butylphenol)	mzc1138	340.24016	12.551	−	−1.84
neg	Glutamic acid	HMDB0000148/HMDB0003339	147.05221	0.603	−	−1.24
neg	Oxidised glutathione	HMDB0003337	612.1527	0.892	−	+1.42
neg	Succinic acid	HMDB0000254	118.02569	0.842	−	+1.17
neg	Uric acid	HMDB0000289	168.0276	0.837	−	+1.70
neg	Uridine	HMDB0000296	244.06954	0.927	−	+1.18
neg	Xanthine	HMDB0000292	152.03263	0.986	−	+1.78
pos	(8)-Shogaol	HMDB0031463	276.1723	8.295	−	+34.32
pos	Bis(4-ethylbenzylidene) sorbitol	mzc7437	414.20382	9.219	+1.48	−
pos	Carnitine	HMDB0000062	161.10519	0.729	−1.24	−1.29
pos	Creatine	HMDB0000064	131.06955	0.655	−1.32	−1.22
pos	Glutathione	HMDB0062697	307.08364	0.766	−3.05	−
pos	Leucine/Isoleucine/Norleucine	HMDB0000687/HMDB0000172	131.09468	0.98	+1.36	−
pos	Niacinamide	HMDB0001406	122.0482	0.67	+1.01	+1.50
pos	Phenylalanine	HMDB0000159/METPA0264	165.07898	1.481	+1.50	−
pos	Pyridoxine	HMDB0000239	169.074	0.853	+1.53	−
pos	Thiamine	HMDB0000235	264.10438	0.77	+1.40	−
pos	Glutamine	HMDB0003423/HMDB0000641	146.06919	0.605	−	+1.25

Abbreviations: MW: molecular weight; RT: retention time; FC: fold change; PSC: pre-senescent control group; PST50: pre-senescent cell treatment group at concentration 50 µg/mL; PST300: pre-senescent treatment group at concentration 300 µg/mL. Symbols: +: increase; −: decrease; neg: negative; pos: positive.

**Table 4:** List of significant metabolites in senescent myoblast groups treated with ginger extract.

Mode	Metabolite	ID	MW	RT	FC	
					ST100 vs. SC	ST300 vs. SC
neg	2,2′-Methylenebis(4-methyl-6-tert-butylphenol)	mzc1138	340.24016	12.551		+3.77
neg	4-Oxoproline	METPA0228	129.04169	0.849	−	−1.88
neg	N-Acetyl-L-aspartic acid	HMDB0000812	175.04736	0.755	−	−1.51
neg	Oxidised glutathione	HMDB0003337	612.1527	0.892	−	+1.73
neg	Uric acid	HMDB0000289	168.0276	0.837	−2.51	−2.58
pos	(8)-Shogaol	HMDB0031463	276.1723	8.295	+16.70	+56.13
pos	Carnitine	HMDB0000062	161.10519	0.729	−1.23	−1.33
pos	Creatine	HMDB0000064	131.06955	0.655	−1.28	−
pos	Cytosine	HMDB0000630	111.04353	0.667	+1.67	−
pos	Glutathione	HMDB0062697	307.08364	0.766	−4.09	−
pos	Octadecanamide	HMDB0034146	283.28734	13.922	+1.85	+2.89
pos	Uracil	HMDB0000300	112.02756	0.932	+1.21	+1.20
pos	Acetylcholine	HMDB0000895	145.1103	0.891	−	−1.26
pos	Adenine	HMDB0000034	135.05467	1.185	−	+36.70
pos	Adenosine	HMDB0000050	267.09665	1.242	−	+45.74
pos	Bis(4-ethylbenzylidene) sorbitol	mzc7437	414.20382	9.219	−	+1.61
pos	Docosanamide	HMDB0000583	339.34976	16.095	−	+3.06
pos	Erucamide	mzc282	337.33412	15.515	−	+2.83
pos	Guanine	HMDB0000132	151.04942	1.274	−	−1.28
pos	Hypoxanthine	HMDB0000157	136.03853	1.254	−	−1.31
pos	Inosine	HMDB0000195	268.08055	1.249	−	−1.35
pos	Oxidised glutathione	HMDB0003337	612.15161	0.9	−	+1.74
pos	Pyroglutamic acid	HMDB0000267	129.04268	0.852	−	−1.79

Abbreviations: MW: molecular weight; RT: retention time; FC: fold change; SC: senescent control group; ST100: senescent cell treatment group at concentration 100 µg/mL; ST300: senescent cell treatment group at concentration 300 µg/mL. Symbols: +: increase; −: decrease; neg: negative; pos: positive.

## Data Availability

The datasets used and/or analysed during the current study are available from the corresponding author on reasonable request.

## Supplementary Materials

The following supporting information can be downloaded at: https://www.mdpi.com/article/10.3390/nu15214520/s1, Figure S1: PCA score plots for young control group (red filled circle) and pre-senescent control group (green filled circle) in negative mode (1a) and positive mode (1b), PCA score plot between senescent control group (green filled circle) and young control group (red filled circle) in negative mode (2a) and positive mode (2b), PCA score plot between young treatment group at concentration 50 (green filled circle) with young control group (red filled circle) in negative mode (3a) and positive mode (3b), PCA score plot between young treatment group at concentration 200 (green filled circle) and young control group (red filled circle) in negative mode (4a) and positive mode (4b), PCA score plot between pre-senescent treatment group at concentration 50 (green filled circle) with pre-senescent control group (red filled circle) in negative mode (5a) and positive mode (5b), PCA score plot between pre-senescent treatment group at concentration 300 (green filled circle) with pre-senescent control group (red filled circle) in negative mode (6a) and positive mode (6b), PCA score plot between senescent treatment group at concentration 100 (green filled circle) with senescent control group (red filled circle) in negative mode (7a) and positive mode (7b), PCA score plot between senescent treatment group at concentration 300 (green filled circle) with senescent control group (red filled circle) in negative mode (8a) and positive mode (8b). Table S1: List of biochemical pathways of significant metabolites profile for myoblast control groups (young, pre-senescent and senescent cells); Table S2: List of biochemical pathways of significant metabolites profile for the young myoblast treated with or without ginger extract; Table S3: List of biochemical pathways of significant metabolites profile for the pre-senescent myoblast treated with or without ginger extract; Table S4: List of biochemical pathways of significant metabolites profile for the senescent myoblast groups treated with or without ginger extract.
