# Supplementary material for: Elucidating the Pharmacological Properties of Zingiber officinale Roscoe (Ginger) on Muscle Ageing by Untargeted Metabolomic Profiling of Human Myoblasts
Source: Nutrients. 2023 Oct 25;15(21):4520. doi: 10.3390/nu15214520 (PMC10648528; doi:10.3390/nu15214520)
Supplement: Supplementary file 1 [file nutrients-15-04520-s001.zip › nutrients-2613012-supplementary.pdf]

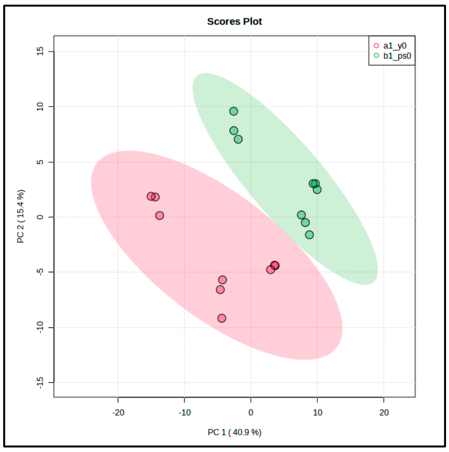

1(a)

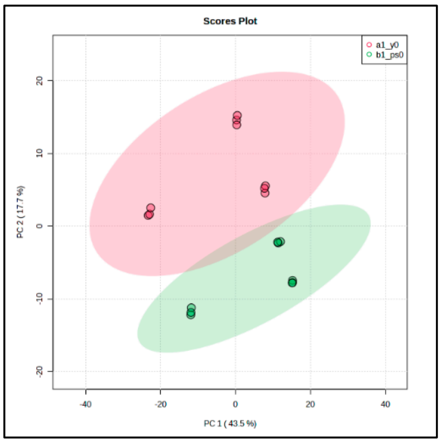

1(b)

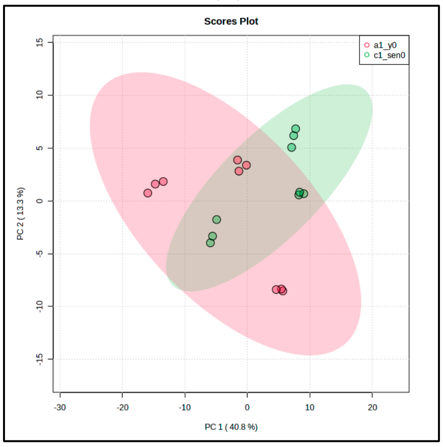

2(a)

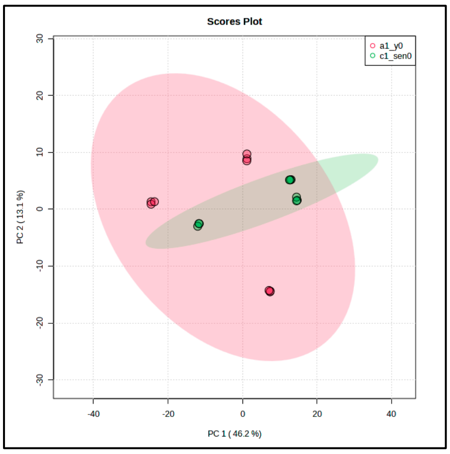

2(b)

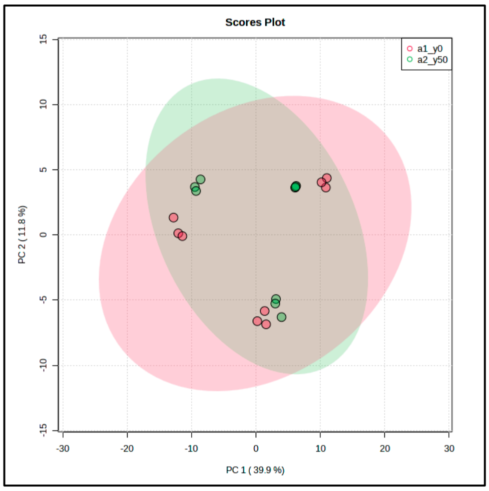

3(a)

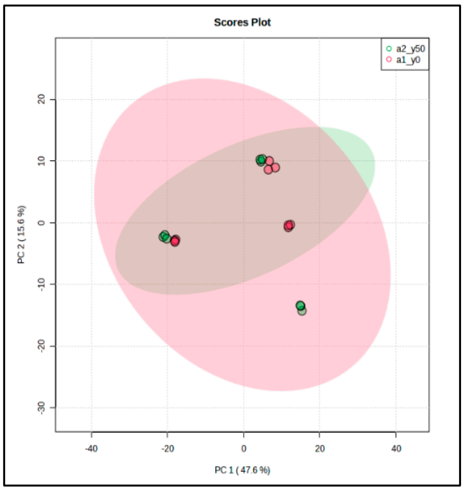

3(b)

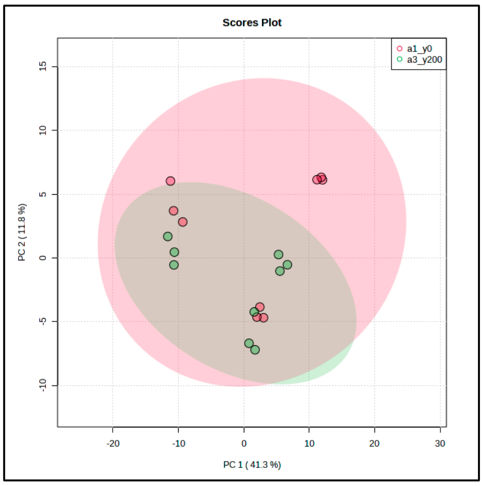

4(a)

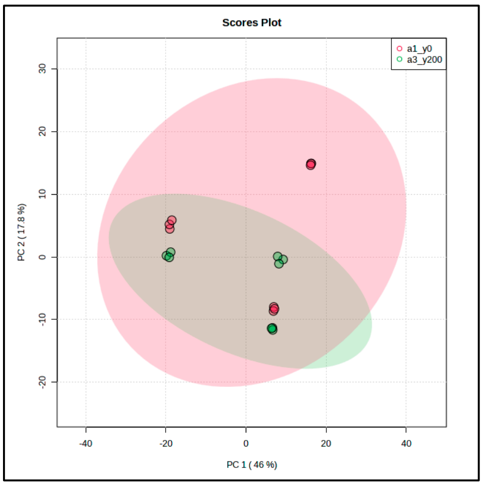

4(b)

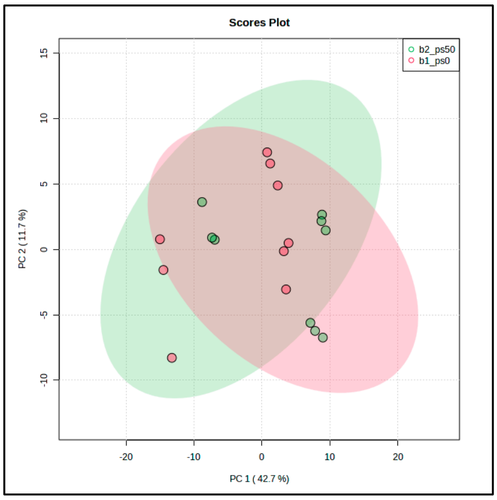

5(a)

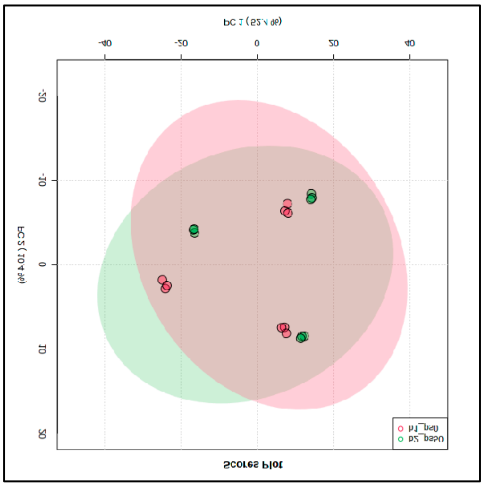

5(b)

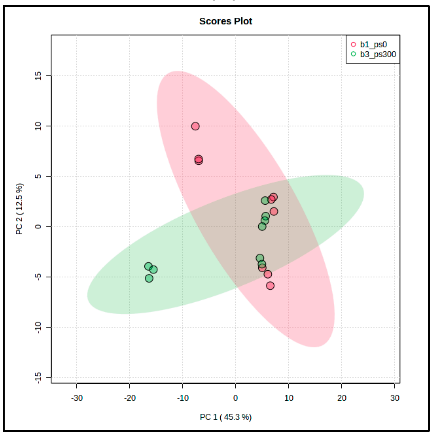

6(a)

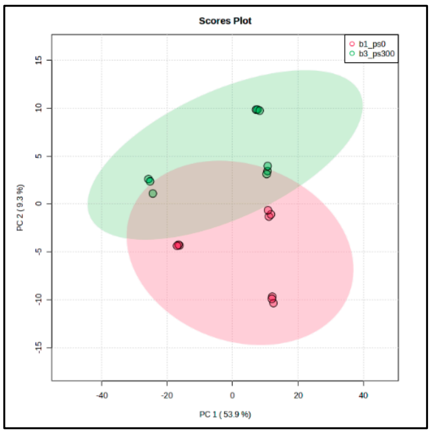

6(b)

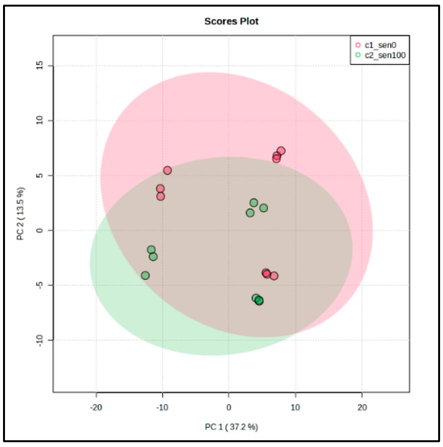

7(a)

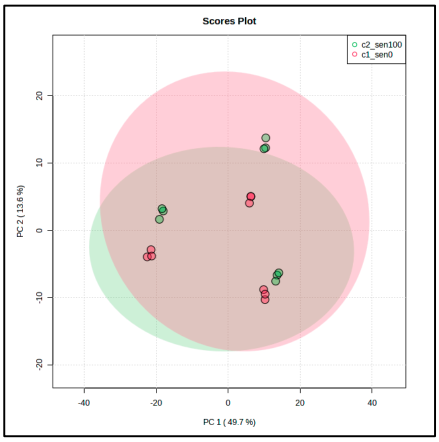

7(b)

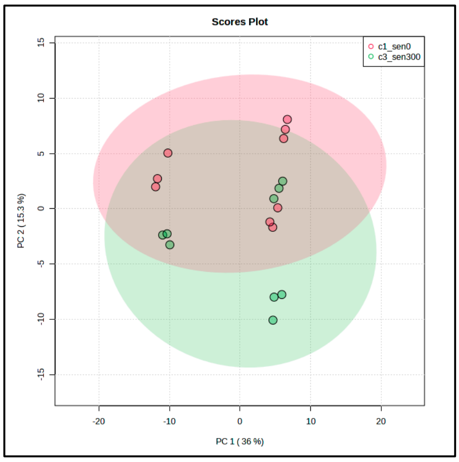

8(a)

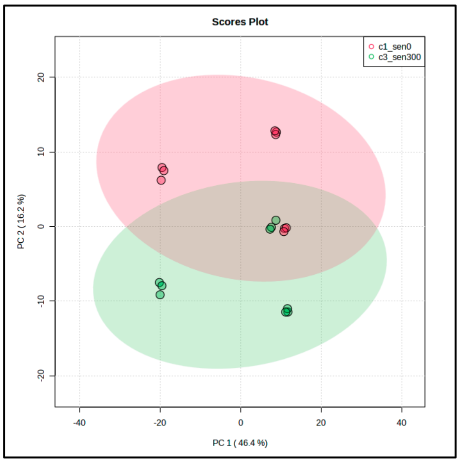

8(b)

Figure 1: PCA score plots for young control group (red filled circle) and pre-senescent control group (green filled circle) in negative mode ((1a)) and positive mode ((1b)), PCA score plot between senescent control group (green filled circle) and young control group (red filled circle) in negative mode (2(a)) and positive mode (2(b)), PCA score plot between young treatment group at concentration 50 (green filled circle) with young control group in negative mode (3(a)) and positive mode (3(b)), PCA score plot between young treatment group at concentration 200 (green filled circle) and young control group (red filled circle) in negative mode (4(a)) and positive mode(4(b)), PCA score plot between pre-senescent treatment group at concentration 50 (green filled circle) with pre-senescent control group in negative (red filled circle) in negative mode (5(a)) and positive mode (5(b)), PCA score plot between pre-senescent treatment group at concentration 300 (green filled circle) with pre-senescent control group (red filled circle) in negative mode (6(a)) and positive mode (6(b)), PCA score plot between senescent treatment group at concentration 100 (green filled circle) with pre-senescent control group (red filled circle) in negative mode (7(a)) and positive mode (7(b)), PCA score plot between pre-senescent treatment group at concentration 300 (green filled circle) with pre-senescent control group in negative (red filled circle) in negative mode (8(a)) and positive mode (8(b)).

Table 1. List of biochemical pathways of significant metabolites profiled for myoblast control groups (young, pre-senescent and senescent cells).

Pathway Name	*p* - value	Impact value
Purine metabolism	8.43E-07	0.05035
Aminoacyl-tRNA biosynthesis	1.26E-05	0.0
**Alanine, aspartate and glutamate metabolism***	**9.78E-05**	**0.39744**
**Phenylalanine, tyrosine and tryptophan biosynthesis***	**0.0018472**	**1.0**
**Arginine and proline metabolism***	**0.0041252**	**0.17592**
Nitrogen metabolism	0.0045154	0.0
**D-Glutamine and D-glutamate metabolism***	**0.0045154**	**0.5**
Glutathione metabolism	0.012788	0.02675
**Phenylalanine metabolism***	**0.012953**	**0.35714**
Glyoxylate and dicarboxylate metabolism	0.018449	0.03175
**Arginine biosynthesis***	**0.02505**	**0.11675**
Butanoate metabolism	0.028584	0.0
Pyrimidine metabolism	0.031243	0.09014
Pantothenate and CoA biosynthesis	0.044529	0.00714
**Citrate cycle (TCA cycle)***	**0.048932**	**0.12311**
Beta-Alanine metabolism	0.053488	0.05597
Glycine, serine and threonine metabolism	0.11788	0.0
Valine, leucine and isoleucine biosynthesis	0.13599	0.0
Glycerophospholipid metabolism	0.13613	0.02582
Ubiquinone and other terpenoid-quinone biosynthesis	0.15168	0.0
Vitamin B6 metabolism	0.15168	0.07843
Nicotinate and nicotinamide metabolism	0.2402	0.1943
Histidine metabolism	0.25406	0.0
Propanoate metabolism	0.34447	0.0
Porphyrin and chlorophyll metabolism	0.42427	0.0
Cysteine and methionine metabolism	0.45553	0.10446
Valine, leucine and isoleucine degradation	0.52222	0.0
Tyrosine metabolism	0.53978	0.13972

*Considered as significant biochemical pathway, p value <0.05 and impact value > 0.1.

Table 2. List of biochemical pathways of significant metabolites profiled for the young myoblast treated with or without ginger extract.

Pathway Name	*P* value	Impact value
**Alanine, aspartate and glutamate metabolism***	**5.83E-04**	**0.20032**
Pyrimidine metabolism	0.024444	0.01584
Aminoacyl-tRNA biosynthesis	0.036071	0.0
D-Glutamine and D-glutamate metabolism	0.038151	0.0
Nitrogen metabolism	0.038151	0.0
Thiamine metabolism	0.044381	0.0
Valine, leucine and isoleucine biosynthesis	0.050574	0.0
Purine metabolism	0.062718	0.01651
Arginine biosynthesis	0.086981	0.0
Butanoate metabolism	0.092925	0.0
Nicotinate and nicotinamide metabolism	0.092925	0.1943
Pantothenate and CoA biosynthesis	0.11636	0.0
Citrate cycle (TCA cycle)	0.12213	0.03273
Propanoate metabolism	0.13924	0.0
Glutathione metabolism	0.16709	0.02698
Glyoxylate and dicarboxylate metabolism	0.18879	0.0
Glycine, serine and threonine metabolism	0.19413	0.0
Arginine and proline metabolism	0.22038	0.01212
Valine, leucine and isoleucine degradation	0.23066	0.0

*Considered as significant biochemical pathway, *p*-value <0.05 and impact value > 0.1.

Table 3. List of biochemical pathways of significant metabolites profiled for the pre-senescent myoblast treated with or without ginger extract.

Pathway Name	*P*-value	Impact value
Aminoacyl-tRNA biosynthesis	7.80E-05	0
**Alanine, aspartate and glutamate metabolism***	**1.34E-04**	**0.3109**
Nitrogen metabolism	0.001464	0
**D-Glutamine and D-glutamate metabolism***	**0.001464**	**0.5**
**Glutathione metabolism***	**0.002528**	**0.3026**
Valine, leucine and isoleucine biosynthesis	0.002699	0
Glyoxylate and dicarboxylate metabolism	0.003732	0.03175
**Arginine biosynthesis***	**0.008461**	**0.11675**
Butanoate metabolism	0.009704	0
**Citrate cycle (TCA cycle)***	**0.017038**	**0.12311**
Purine metabolism	0.02666	0.01217
**Phenylalanine, tyrosine and tryptophan biosynthesis***	**0.040694**	**0.5**
Arginine and proline metabolism	0.056589	0.09812
Pyrimidine metabolism	0.059292	0.01584
Valine, leucine and isoleucine degradation	0.062041	0
Thiamine metabolism	0.07019	0
Vitamin B6 metabolism	0.08938	0.07843
Phenylalanine metabolism	0.098834	0.35714
Nicotinate and nicotinamide metabolism	0.14474	0.1943
Histidine metabolism	0.15366	0
Pantothenate and CoA biosynthesis	0.17988	0
Propanoate metabolism	0.21367	0
Porphyrin and chlorophyll metabolism	0.26966	0

*Considered as significant biochemical pathway, p value <0.05 and impact value > 0.1.

Table 4. List of biochemical pathways of significant metabolites profiled for the senescent myoblast groups treated with or without ginger extract.

Pathway Name	*P*-value	Impact value
Purine metabolism	4.49E-07	0.03818
**Glutathione metabolism***	**0.001684**	**0.29003**
**Alanine, aspartate and glutamate metabolism***	**0.02505**	**0.20032**
Pyrimidine metabolism	0.046401	0.0743
D-Glutamine and D-glutamate metabolism	0.053068	0
Nitrogen metabolism	0.053068	0
Arginine biosynthesis	0.11976	0
Pantothenate and CoA biosynthesis	0.1592	0
beta-Alanine metabolism	0.17452	0
Glyoxylate and dicarboxylate metabolism	0.2542	0
Glycine, serine and threonine metabolism	0.26108	0
Glycerophospholipid metabolism	0.28136	0
Arginine and proline metabolism	0.2946	0.01212

**Pathway Name**	***P*-value**	**Impact value**
Aminoacyl-tRNA biosynthesis	0.35744	0

Considered as significant biochemical pathway, p value<0.05 and impact value > 0.1.
